# Supplementary material for: Tannic acid, an IL-1β-direct binding compound, ameliorates IL-1β-induced inflammation and cartilage degradation by hindering IL-1β-IL-1R1 interaction
Source: PLoS One. 2023 Apr 20;18(4):e0281834. doi: 10.1371/journal.pone.0281834 (PMC10118179; doi:10.1371/journal.pone.0281834)
Supplement: S1 Table — (DOC) [file pone.0281834.s001.doc]

**Table S1.** **RT-qPCR primer sequences used in the present study**

| **Gene** | **Primer sequences** | | **Amplicon length (bp)** |
| --- | --- | --- | --- |
|  | **Forward (5’-3’)** | **Reverse (5’-3’)** |  |
| *GAPDH* | CGA GAT CCC TCC AAA ATC AA | TTC ACA CCC ATG ACG AAC AT | 170 |
| *NOS2* | CCT TAC GAG GCG AAG AAG GAC AG | CAG TTT GAG AGA GGA GGC TCC G | 278 |
| *COX-2* | GAG AGA TGT ATC CTC CCA CAG TCA | GAC CAG GCA CCA GAC CAA AG | 117 |
| *IL-6* | TAC CCC CAG GAG AAG ATT CC | TTT TCT GCC AGT GCC TCT TT | 175 |
| *TNF* | CCC AGG GAC CTC TCT CTA ATC | ATG GGC TAC AGG CTT GTC ACT | 84 |
| *MMP3* | TGA GGA CAC CAG CAT GAA CC | ACT TCG GGA TGC CAG GAA AG | 248 |
| *MMP13* | CTG GCC TGC TGG CTC ATG CTT | GCA GGG TCC TTG GAG TGG TCA | 166 |
| *COL2A1* | TGG ACG ATC AGG CGA AAC C | GCT GCG GAT GCT CTC AAT CT | 244 |
| *ACAN* | GGG TCA ACA GTG CCT ATC AG | GGG TGT AGC GTG TAG AGA TG | 213 |
| *ADAMTS4* | CCT GGC AAG GAC TAT GAT GCT GA | GGG CGA GTG TTT GGT CTG G | 150 |
| *ADAMTS5* | GCA GAA CAT CGA CCA ACT CTA CTC | CCA GCA ATG CCC ACC GAA C | 114 |
