# Supplementary material for: Tannic acid, an IL-1β-direct binding compound, ameliorates IL-1β-induced inflammation and cartilage degradation by hindering IL-1β-IL-1R1 interaction
Source: PLoS One. 2023 Apr 20;18(4):e0281834. doi: 10.1371/journal.pone.0281834 (PMC10118179; doi:10.1371/journal.pone.0281834)
Supplement: S1 Raw images — (PDF) [file pone.0281834.s002.pdf]

Figure 3B Effects of TA on the expression of ADAMTSs, COL2A1 and ACAN in IL-1β-stimulated human OA chondrocytes

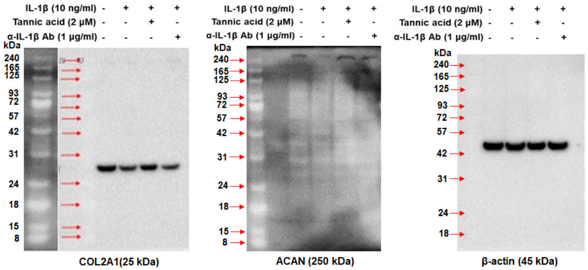

Figure 3B Effects of TA on the expression of MMP3 and MMP-13 in IL-1β-stimulated human OA chondrocytes

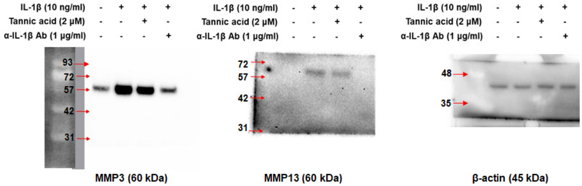

Figure 4A Effects of TA on IL-1β-induced MAPK activation in human OA chondrocytes

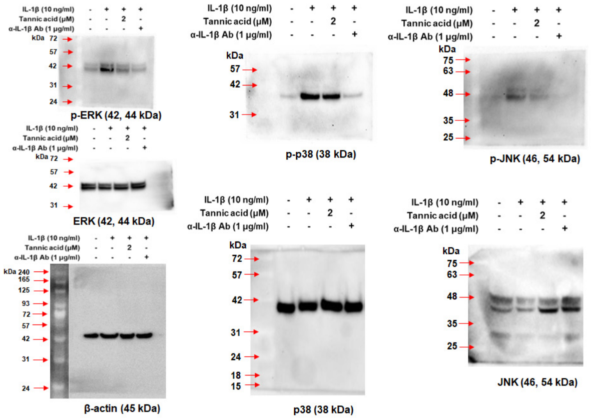

Figure 4B Effects of TA on IL-1β-induced NF-κB activation in human OA chondrocytes

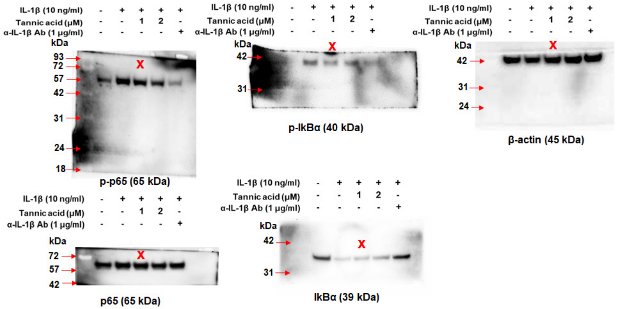

Any lane with X symbol is not represented in the figure submitted with in the manuscript
